# Exploring faculty perception of simulation-based education: Benefits and challenges of using simulation for improving patient safety in cardiovascular diploma program

**DOI:** 10.12669/pjms.39.2.6693

**Published:** 2023

**Authors:** Ajlan A Alshehri, Fahaad S Alenazi, Hamad Alturki, Farida Habib Khan

**Affiliations:** 1 Dr. Ajlan A Alshehri, King Fahad Medical City, Kingdom of Saudi Arabia; 2 Dr. Fahaad S Alenazi, Department of Pharmacology, Medical Education Unit, College of Medicine, University of Ha’il, Kingdom of Saudi Arabia; 3 Dr. Hamad Alturki, Department of Medical Education, College of Medicine, Imam Mohammed Ibn Saud Islamic University, Kingdom of Saudi Arabia; 4 Prof. Dr. Farida Habib Khan, Dept. of Family & Community Medicine, Dept. of Medical Education, College of Medicine, University of Ha’il, Kingdom of Saudi Arabia

**Keywords:** Allied healthcare education, Cardiac diploma, Curriculum, Faculty-development, Patient safety, Simulation

## Abstract

**Objectives:**

The study was conducted in a tertiary educational hospital based in Riyadh to explore faculty’s perception of using simulation-based teaching as part of the Cardiovascular Diploma Program (CDP) to improve patients’ safety. The study, also aimed to identify the benefits and challenges of utilizing simulation.

**Methods:**

Researchers used a qualitative approach. Semi-structured interviews were conducted online with ten faculty-members. The interviews were performed between July and September in the year 2019. Authors used convenient sampling techniques for recruitment. Data were transcribed and analyzed using a framework analysis approach.

**Result:**

Data analysis showed four emergent themes, i.e., the concept of simulation (it is a risk-free environment for training), simulation for patients’ safety (students first learn on the simulators and deal with patients), simulation as a safe learning environment (gives idea basic things about the working environment, knowing the symptoms of the patients, catheterizing the patient, knowing preparations for the procedure and post care), and the challenges of utilizing simulation (identify gaps between the theoretical and practical parts).

**Conclusion:**

Faculty has appreciated the role of simulation in improving patients’ safety. Simulation was underutilized due to the limited time allotted for simulation and lack of adequate experienced faculty. It is recommended that simulation should be integrated into the CDP curriculum.

## INTRODUCTION

Simulation is utilized in different areas in healthcare institutions. In 85% of skills development programs, simulation is practiced.^1^ It is used to improve patients’ safety and enhance healthcare education.^1^ Simulation-based education provides trainees an opportunity to practice practical cases in a safe learning and teaching environment.^2^,^3^ Importantly, simulation ties the knowledge to practice and grooms the healthcare trainees to be safe and efficient in the clinical environment.

Thus, many studies have stressed on the use of simulation-based education for patients’ safety. Eide et al. studied novice students’ handover skills using simulation scenarios and practice.^4^ They found that simulation could prepare students for future work and improve patient’s safety. Furthermore, simulation offers an appropriate environment for learning, helping students enhance their skills by repeated practice and learning from mistakes without consequences to themselves and, more importantly, to the patients. Additionally, the capability to receive direct and immediate feedback is another beneficial hallmark of simulation, which may not always exist in the clinical workplace. Studies reported that teaching with simulators enhanced learning outcomes regarding diagnosis and management.^5^,^6^ Benefits associated with the simulation use in clinical setting have encouraged medical colleges to initiate its use in medical curricula.^5^ Therefore most healthcare training programs have incorporated simulations into their curriculum.

In fact, simulation is widely used in medicine and nursing curricula but is considered relatively recent to the allied healthcare professions in Saudi Arabia. Nevertheless, there are barriers in using simulation in healthcare programs. A study done by Ahmed et al. states that lack of experienced simulation specialists and the high cost of simulators creates barriers to simulation utilization.^6^ Also, Al-Ghareeb & Cooper, highlighted that time constraints for simulation and sophisticated simulators are considered barriers to simulation utilization.^7^ These barriers are a result of different research circumstances and areas. However, it could be different from the perspective of the cardiovascular faculty.

Hence, the authors would like to understand the cardiovascular program’s faculty viewpoints about simulation utilization as an educational method to improve patient’s safety and explore the usefulness of simulation and its barriers from the perspective of the program faculty. The aforementioned issues were poorly investigated in the context of the allied healthcare program.^8^,^9^ Author believes that the study will open the door for the researchers to thoroughly explore these issues and provide solutions

The study aimed to explore the faculty’s opinion of using simulation-based education to improve patients’ safety in the CDP at tertiary hospitals in Saudi Arabia and identify the benefits and challenges of using simulation to educate cardiovascular students.

## METHODS

### Conceptual orientation Research design:

The study was designed based on the phenomenological methodology to explore people’s opinions and experiences. Thus, a qualitative design was utilized to explore faculty’s opinion about simulation as part of the cardiovascular curriculum to improve patient safety.

### Setting and context:

This study reviewed a Cardiovascular Diploma Program (CDP) available at a prominent institute in Saudi Arabia. This program has been delivered in two years. First-year classes include introductory modules, whereas second-year classes are devoted to core modules. The curriculum had no space for simulation training sessions, and most of the training has occurred in the Cardiac Catheterization Laboratory (CCL) environment.

### Ethical Considerations:

Institute Review Board, King Fahad Medical City, Riyadh, KSA, approved the study (IRB Log Number:19-321E). Invitations were sent to the candidates embedded with the consent and the interview information confidentiality forms. Additionally, the candidates were informed about the study goal, method, impact of the study, and their rights as participants. The consents were signed and returned to the author, confirming participation in the study.

### Sampling and recruitment:

Study population included the faculty involved in teaching CDP students. The study utilized convenient sampling techniques to recruit interviewees. The sample size of the study was twenty-four participants. The participants in the interviews were coded as Interviewee-1, Interviewee-2, Interviewee-3, and so on.

### Data collection:

This study was conducted from July to September in the year 2019. It was based on a series of one-to-one semi-structured interviews facilitated by the author. The interview questions were designed and crosschecked with the supervisor and peers in the medical education center. Interviews’ average time was about 30 minutes. All interviews were recorded and saved in a shared file with the supervisor. However, remarks have been taken during the interview as a backup.

### Data analysis approach:

Resulting data was analyzed according to the framework approach, focusing on the words and phrases related to the research objectives. The first step of the analysis was transcribing the interviews. The transcribing was carried out in two phases, the first round was after the first three participants, and the second round was for the last three participants. A small portion of the transcription included a translation from Arabic to English, which is common in interviews transcription.^9^,^10^ Author has listened to the interviews’ records and read the transcripts many times to elicit the ideas and themes. These ideas and themes were documented in an excel sheet for deep analysis and understanding of the phenomena. Then, the author identified the main themes aligned with the study goal.

### Quality and rigor:

The study’s trustworthiness was checked to ensure its credibility, dependability, transferability, and conformability. The author deliberated the transcripts and the study findings with the medical educationist and peers in medical education. Moreover, the research findings were triangulated with the literature of a similar context, as this study can support other studies with approximate research context.

## RESULTS

**Fig.1:**
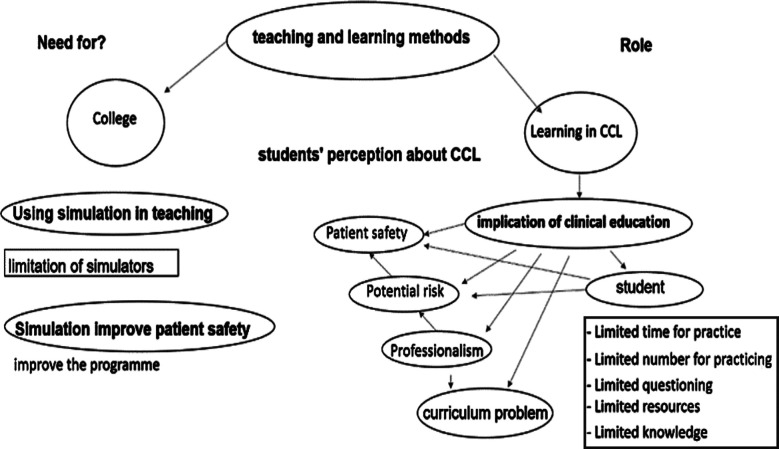
Multifaceted Ideas.

The first version of the themes was multifaceted because vast themes were generated from the first twenty interviews (Fig.1). The data was saturated after the last four interviews; most of the latest data were repeating the previous interviews. Considerable time was spent linking some emergent themes to have broad themes with constructed meaning aligned with the study objectives.

**Fig.2:**
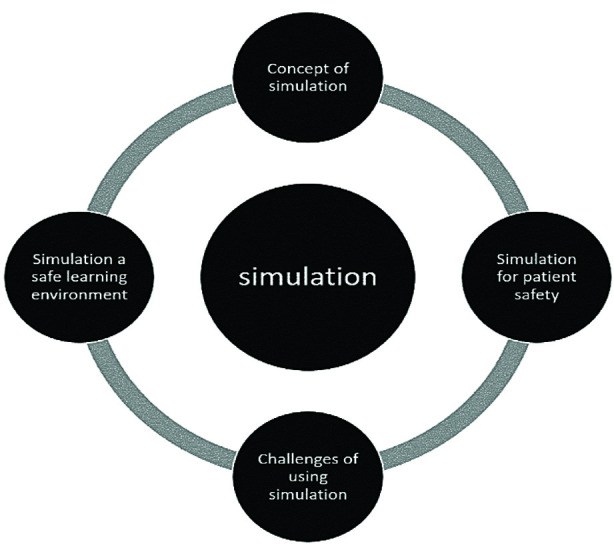
Emerged Themes.

The raw data uncovered four emergent themes: the concept of simulation, the benefit of simulation for patients’ safety, simulation as a safe learning environment, and challenges of using simulation (Fig.2).

### Concept of simulation:

The faculty understood the simulation concept, which replicated an actual situation away from the patient and in an area free of risk.

“Simulation mainly is for training” (Interviewee-1). “It is a risk-free environment for training” (Interviewee-2). “You can teach your students without risk of radiation, without putting the patient in danger and give them a very close environment to the real Cath. Lab without taking any hazards or risk that you take in the real environment” (Interviewees 3 & 23).

The faculty stated that simulation is a method that helps them to educate the CCL team on cardiac cases and reduce healthcare errors.

“Simulation is devices and areas for training and getting experience about the cardiac cases and teamwork in CCL” (Interviewee-4). “It is beneficial for teaching and learning” (Interviewee 5). It reduces the errors and minimizes mistakes that may happen during the procedure, especially resulting from a lack of communication skills” (Interviewees-6 & 22).

### The benefit of simulation for patient safety:

Faculty understood that the risk to patient increases with novice students:

’If we draw a curve between the risk and the stage of training, whenever the stage is early, the risk is high” (Interviewee-7). “There is a proportional relationship between the risk and the stage of learning (Interviewees-9 & 23).

Faculty believed that simulation is an effective tool to improve patient safety in the cardiovascular program because they will be equipped with needed skills.

“The simulation will give students a clear step with practice; the student will easily understand through simulation” (Interviewees-20 & 24). “Student can learn and be able to analyze the arrhythmia as an example. “Simulation based training, definitely, will help to improve patient safety” (Interviewee-11). “Effective tool for knowledge retention and to enhance the knowledge retention and improve the psychomotor skills, especially in the cardiovascular care where the errors or malpractice can generate a catastrophic or fatal effect” (Interviewees-4 & 21).

The faculty accentuated that simulation can be used as a transitional media from academic to clinical environment. It can improve cardiovascular students’ confidence by preparing them for the clinical part.

“I believe simulators will offer great help, especially if you are going to deal with a human; you want the students to learn first on the simulators and deal with it” (Interviewee-12). “Students will be more confident when they deal with the patient; it will be strength for them. It is a transition stage when they need to go through first to enable the student to deal with the patient. So, simulators will provide great help” (Interviewee-4).

’They can practice [in simulation], and when they come to the Cath. Lab, they will be more than half-prepared and ready to take over’ (Interviewee-14).

### Simulation as a safe learning environment:

Faculty thought that educating the students in a simulation might provide them with a basic understanding of what is CCL and the workflow atmosphere:

’For education, simulation is very important because it gives them [the students] ideas or basic things about the working environment, starting from knowing the symptoms of the patients, catheterizing the patient, what are the preparations for the procedure, the procedure and post-procedure” “ (Interviewees-1 & 15). “I think it will be a great tool for introduction to the procedure area, especially for catheterization” (Interviewees-16 & 25).

The faculty pointed out that simulation overcomes the difficulties of traditional training. It reduces the gap of knowledge and practice. Also, it eases the transferring of knowledge between the trainer and trainee.

“The learning process without simulation was slow because the students would not have full access to tools and materials found in the simulation [in the traditional method] I would draw for the students, which was far from the real-life experience” (Interviewee-18). “With the simulation, that was a game-changer, as you can do many variations with it, it is easy to create, to play, to change, and do many things with it, you can play as an actual life event for the students. It [traditional method] was time-consuming producing the materials without the simulation. With simulation, you can get excellent results for the students there” (Interviewees-19 & 24).

### Challenges of utilizing simulation:

Faculty indicated that simulation could help students apply what has been taught in classrooms, bridging the gap between theory and practice. However, they did not utilize it sufficiently in the cardiac program.

“I believe that simulation is a great tool but underused up-to-date. I think simulation could link between the theoretical and practical parts. Simulation enables the student to feel stents, wires, and CCL stuff, in reality, touch it and live the experience” (Interviewee-9).

Faculty stated that simulation could enhance the teaching methods; however, they did not use it because of a shortage of trained faculty on simulation. Also, they believed that there is a need for qualified healthcare educators.

“Simulation is an asset; unfortunately, we failed to use it is a good environment to teach students, but we lack the resources like education staff” (Interviewees 10 & 23).

“For simulation, challenges are finding qualified teachers” (Interviewee 6).

Interviewees think that simulators are expensive with limited features specifically related to hemodynamic.

’The market concentrates on simulators that use catheters, wires, and other equipment, but not the hemodynamic changes” (Interviewee 5). Until now, we did not have a simulator with a concentration on the hemodynamic. Also, it needs a high budget to have a simulator’ (Interviewees 9 & 25).

## DISCUSSION

Conversing with the faculty has exposed their critical thinking about the utilization of simulation to improve patient safety and the educational environment in the CDP context. It has emerged important themes: (1) the concept of simulation, (2) simulation benefits for patient safety and (3) healthcare professional education, and (4) challenges of using simulation.

### Concept of simulation:

Faculty showed an understanding of simulation as a tool to bridge the gap between theory and practice, which mainly reflects Gaba’s definition of simulation as replicating actual situations as notified by Newell and Doorey.^10^,^11^ However there was confusion about the perception of simulation. They thought that simulation is mannikin, which is flawed because it is a technique, not a device that simulates actual situations. They can use simulation to improve soft skills such as communication and leadership as notified by Henien.^12^

Unlike hospital wards, CCLs are complex, dynamic, and challenging workplaces, where mistakes can have severe consequences for patients and employees.^13^ The CCL quality team strives to lower the risks and improve patient safety.^14^ Interrupting or distracting the team members can disrupt the communication channel and put patients at risk.^15^ Since novices tend to ask questions to get an understanding, they could pose a threat to the safety of CCL patients because their questions can disrupt the communication channel.^16^ In addition, they could prolong the time of the procedure; increase radiation time, and contrast media dosage.^15^,^17^ Moreover, Faggioni et al., found that healthcare students had a weak understanding of radiation safety in radiated areas, which could harm them.^16^

### The benefit of simulation for patient safety:

The main goal of the training in CCL was to consolidate some gained knowledge, learn technical skills, and perform teamwork.^18^ Still, these were a detriment of the workflow in the CCL, affected the outcomes, and compromised patient safety.^19^ The CDP tutors should use simulation to teach cognitive, psychomotor, and communication skills to individuals or groups of students to boost patient safety, as identified by Motola et al. in 2013.^17^ Furthermore, underline the importance of simulation to teach essential clinical skills, such as teamwork and communication, which may not be easy to show in CCL because they possibly break the communication between the CCL staff and cause an undesirable situation to the patient safety.^20^ Simulation could provide enough training time for students and prepare them for the program’s clinical part. So, when students work in the CCL, they know how to minimize, or avoid, putting patients at risk.

The faculty also emphasized simulation’s effect on minimizing errors through continuous training in a safe environment with no harm to the patient. These findings agreed with Motola et al.^17^ Also, the findings aligned with those of Goldacre et al.^18^ as well as Ker & Bradley^2^ in that simulation replicated an actual cardiac situation, which enriches the students’ minds by analyzing problems and learning to take action.

Faculty asserted simulation use for educational purposes to improve patients’ safety and reduce the negative influence on students, same findings were reported by Shiner and Munshi .^21^,^22^ The study of Lefor et al. confirmed the use of simulation to push healthcare education toward patient-centered care to improve patient safety.^23^ In addition, Lane and Bambini et al., verified that simulation-based healthcare education enhanced students’ self-efficacy and practical skills, which positively boosted patient safety.^24^,^25^ Moreover, Issenberg et al.^5^, and Lane et al.^24^ added that simulation could minimize students’ learning curve and introduce well-prepared learners to health care. Furthermore, patients feel safe and they can accept the presence of the students if they are acting confidently and skillfully.^21^,^22^

### The benefit of simulation for healthcare education:

Unlike hospital wards, CCLs are complex, dynamic, and challenging workplaces, where mistakes can have severe consequences for patients and employees.^10^ The CCL quality team strives to lower the risks and improve patient safety.^11^ Communication in this dynamic workplace is crucial. Interrupting or distracting the team members can disrupt the communication channel and put patients at risk.^12^ Since novices tend to ask questions to get an understanding, they could pose a threat to the safety of CCL patients because their questions can disrupt the communication channel.^13^ In addition, they could prolong the time of the procedure; increase radiation time, and contrast media dosage.^12^,^15^ Moreover, Faggioni et al., found that healthcare students had a weak understanding of radiation safety in radiated areas, which could harm them.^16^ Accordingly, students can learn non-technical skills at simulation and enhance other competencies.

### The benefit of simulation for patient safety:

The faculty highlighted the simulation’s critical features in improving the students’ clinical skills and patient safety. The main goal of the training in CCL was to consolidate some gained knowledge, learn technical skills, and perform teamwork. Still, these were a detriment of the workflow in the CCL, affected the outcomes, and compromised patient safety.^20^ The CDP tutors should use simulation to teach cognitive, psychomotor, and communication skills to individuals or groups of students to boost patient safety, as identified by Motola et al., in 2013.^17^ Furthermore, underline the importance of simulation to teach essential clinical skills, such as teamwork and communication, which may not be easy to show in CCL because they possibly break the communication between the CCL staff and cause an undesirable situation to the patient safety. Also, students do not have sufficient time for training in CCL as their training time is bounded. Thus, the simulation could provide enough training time for students and prepare them for the program’s clinical part. So, when students work in the CCL, they know how to minimize, or avoid, putting patients at risk.

The faculty also emphasized simulation’s effect on minimizing errors through continuous training in a safe environment with no harm to the patient. These findings agreed with Motola et al.^17^ Also, the findings aligned with those of Goldacre et al.^18^ as well as Ker & Bradley^2^ in that simulation replicated an actual cardiac situation, which enriches the students’ minds by analyzing problems and learning to take action.

Faculty asserted simulation use for educational purposes to improve patients’ safety and reduce the negative influence on students. The study of Karakoc A et al. confirmed the use of simulation to push healthcare education toward patient-centered care to improve patient safety.^3^ In addition, Krathwohl verified in his article on Bloom’s Taxonomy that simulation-based healthcare education enhanced students’ self-efficacy and practical skills, which positively boosted patient safety.^19^ Moreover, Naidu et al.^15^, and Faggioni et al.^16^ added that simulation could minimize students’ learning curve and introduce well-prepared learners to health care. Furthermore, patients feel safe and they can accept the presence of the students if they are acting confidently and skillfully.^17^

### The benefit of simulation for healthcare education:

Clinical educators in health care are focusing on patient safety. Therefore, in the CDP, the faculty pointed out that establishing education in regular classrooms is fundamental in the early stage of the program, which is agreed with Bloom’s Taxonomy.^19^ Bloom’s classification identifies six cognitive domains: knowledge, comprehension, application, analysis, synthesis, and evaluation. A gradual introduction to learning from the lower level of the taxonomy to the upper level, with no jump at any level, is essential because each level provides a deep understanding of the following level as stated by Krathwohl.^19^ However, the CDP curriculum was designed to let students attend the CCL in an early stage, which is not aligned with Bloom’s taxonomy. Therefore, it is appropriate to start educating students in classrooms while the patient is not part of their practice, and that was what faculty has thought.

Previous studies have shown that simulation-based skill learning showed significantly better results (p=0.05) as seen through performance in OSCE. Further, it is evident from a study done in Pakistan, that simulation-based medical education is evidence-based teaching learning modality.^20^

However, one of the drawbacks of this method is simulation-based education requires hands-on teaching and therefore only limited number of students can be accommodated at a particular time.^19^,^20^ Thus, the author believes that consulting faculty about the best way of training as per their area of expertise is likely to improve the curriculum and patient safety.

The faculty agreed that simulation provides a safe learning environment for students. According to Maslow’s motivational theory, the student who feels insecure will not be motivated to learn. So, safety is an essential part of stimulating students’ learning in the CCL, and that might not be the case when the students lack knowledge and experience. Thus, simulation can prepare students psychologically for CCL work. Shiner found that moulage simulation minimized the harmful psychological effects of what could be seen in the clinical site, such as patient wounds, disturbance, and excitement.^21^ Students in the CCL can see patients’ blood and injuries, so it is vital to expose them safely before introducing them to cardiac catheterization laboratories’ patients, and that was what the faculty emphasized. Although faculty recognized that simulation is a safe area for educating novice students, they do not know how to achieve it.^22^ Therefore, the author believes that medical educationist experts should revise any healthcare curriculum to identify where simulation can fit.

### Challenges of using simulation

*The primary barriers raised by the CDP faculty were*:

- The cost of the simulators.
- The features of the available cardiac simulators.
- The fidelity of the simulators.

The simulators’ cost increases with increased feature, and the price and features of the simulator depend on the companies.^23^,^24^ So the discussion concentrated on the fidelity of the simulator rather than the price and the features.

The fidelity of simulation is the degree to which it mimics real life. The findings agreed with a study where, students’ engagement depends more on how much they believed in the simulation.^21^ Moreover, Munshi et al., supported the importance of selecting an appropriate level of simulation fidelity based on a learner’s level.^22^ However, the students’ engagement in simulation depends on the faculty, and fidelity should not be the main target of education and training.^23^ In fact, faculty should expedite low- and high-fidelity simulation to enhance the educational techniques as reported by Bambini as well. 25

Unfortunately, faculty has lacked simulation experience, which often drives them into the trap of fidelity. For instance, they think an x-ray simulator should work as a real machine, which is impossible in the meantime. That could be the reason for the limited use of simulation in the CDP. These findings were supported by the studies done in Taibah University, which indicated that 54% of faculty lack training and part of the training should be on simulation.^22^

Train the faculty to utilize simulation intelligently and improve students’ engagements. The study done by Lefor et al., supported the need to enlighten the faculty about simulation fidelity and suggested tips to alleviate simulation realism problems.^23^ For example, the faculty should establish a fictitious contract with students by admitting that the simulators are not accurate but used to achieve specific objectives. This contract can help students accept the level of fidelity and concentrate on the simulation activity’s goal as reported by Shafiq from a study done in Pakistan.^26^ Also, it can minimize the cost of the simulation because faculty can work according to a limited budget. Thus, it is vital to prepare faculty to use simulation efficiently.

It is proven in many national and internal research studies that simulation-based medical education is evidence-based teaching learning modality that enhances competency of trainees.^23^,^26^ Hence, Simulation-Based Medical Education has become cornerstone in medical education for teaching technical and non-technical skills in a safe, non-threatening, and controllable environment as reported in studies done in Pakistan.^27^,^28^

### Limitations:

The study was conducted in one institute, limiting the generalizability of the study’s findings. However, the study may also be a lens to highlight the same issues of implemented simulation-based education in other centers in Saudi Arabia or beyond. Also, significant stakeholders – students and patients – were not involved in this study. Thus, future research might include more than one center and include students and patients in the study.

## CONCLUSION

The faculty confirmed the advantages of simulation-based education in enhancing patient safety. However, the faculty had a superficial understanding of simulation. They lacked the experience to exploit the available resources of simulation. Thus, it is essential to consult a medical educationist or certified simulation specialist on the best methods to embed simulation in the curriculum based on the available resources.

The study results would be helpful for medical educationist while designing medical curricula, as well as to the trainers of clinical skills. The use of clinical simulations in medical education is often referred to as performance-based learning. The study found that, this methodology allows trainers to identify, solve and reflect on their performance by using simulation techniques as part of the teaching and evaluation curriculum of clinical skills. Working in a simulated environment allows learners to make mistakes without the need for intervention by experts to stop patient harm. By seeing the outcome of their mistakes, learners gain powerful insight into the consequences of their actions. Simulation provides standardization of cases, promotes critical thinking, allows supervision of patient care, provides immediate feedback, and helps students to assimilate knowledge and experience.

Hence the authors recommended train-the-trainer courses for the faculty, including developing a curriculum, available simulators, and features to optimize its use. The faculty should be motivated by rewards and free time to provide the best of what they have. Additionally, the vendors and manufacturers of simulators should consider simulators’ prices and specifications affordable to healthcare users.

### Authors’ Contributions:

**AA, FA & HA:** Conceived and designed the study.

**AA, FA & HA:** Collected the data and performed the analysis.

**AA, HT & FH:** Were involved in the writing of the manuscript and are responsible for the integrity of the study.

All authors have read and approved the final manuscript.
